# Efficacy of a high-potency multivalent foot-and-mouth disease virus vaccine in cattle against heterologous challenge with a field virus from the emerging A/ASIA/G-VII lineage

**DOI:** 10.1016/j.vaccine.2018.02.016

**Published:** 2018-03-27

**Authors:** Ryan Waters, Anna B. Ludi, Veronica L. Fowler, Ginette Wilsden, Clare Browning, Simon Gubbins, Bob Statham, Abdelghani Bin-Tarif, Valerie Mioulet, David J. King, Claire Colenutt, Emma Brown, Pascal Hudelet, Donald P. King

**Affiliations:** a The Pirbright Institute, Ash Road, Pirbright, Woking, Surrey GU24 0NF, United Kingdom; b Boehringer Ingelheim, 29 Avenue Tony Garnier, 69007 Lyon, France

**Keywords:** Foot-and-mouth disease virus, A/ASIA/G-VII, Podal generalisation test (PPG), Challenge, Vaccine

## Abstract

In 2015, outbreaks of foot-and-mouth disease (FMD) in the Middle East were discovered to be caused by a viral lineage (A/ASIA/G-VII), which has recently emerged from the Indian sub-continent. *In vitro* vaccine matching data generated by the World Reference Laboratory (WRLFMD) indicated that A/ASIA/G-VII field viruses were poorly matched with vaccines (A-SAU-95, A22 IRQ and A-IRN-05) that are already used in the region. In order to assess the likely performance of one of these commercially available FMD vaccines, sixteen cattle were vaccinated with a polyvalent vaccine which contained two serotype A components (A-SAU-95 and A-IRN-05) with a homologous potency of at least 6PD_50_, and two cattle were left unvaccinated as controls. Twenty-one days later, all 18 cattle were challenged by tongue inoculation with an FMDV field isolate A/IRN/22/2015 from the A/ASIA/G-VII lineage, in line with the European Pharmacopeia PPG test conditions. The two control animals developed generalised FMD, and 7/16 vaccinated animals developed at least one foot lesion, thus only 56.3% were defined as protected. For the vaccine components, there was a significant increase in the probability of protection with increasing serological titres for A-SAU-95 (p = 0.03), but not for A-IRN-05 (p = 0.42). Analysis of FMDV in blood and nasal swabs suggested that vaccination reduced shedding and potential onward spread of FMD virus even if the animal developed foot lesions. In summary, the results from this study suggest that whilst this vaccine would not be appropriate for use in an emergency situation (in previously FMD-free countries), it may be partially effective in the field in endemic countries where repeat prophylactic vaccination is practiced. For emergency reactive vaccination, the findings from this study support the idea that a new vaccine strain should be developed that is tailored to the A/ASIA/G-VII lineage.

## Highlights

• FMDV A/ASIA/G-VII lineage has recently spread beyond the Indian sub-continent.
• Study evaluated the performance of a high potency polyvalent vaccine in cattle.
• A new vaccine strain should be developed which is tailored to the A/ASIA/G-VII lineage.

## 1 Introduction

Foot-and-mouth disease (FMD) is one of the most economically important diseases of livestock, largely due to the direct production losses in endemic areas [1], constraints on international trade, and costs to control the disease [2]. FMD virus (FMDV), the aetiological agent responsible for FMD, is a positive-sense, single stranded RNA virus. Seven distinct serotypes of FMDV have been described; initially defined in terms of their ability to elicit cross protection after infection, and now described largely in terms of *in vitro* serological cross reactivity. Within each of these serotypes, antigenically distinct viruses can occur that are defined by serological and genetic methods.

In FMD free countries, FMD is controlled by restricting animal movements, culling infected animals, and enhancing/implementing farm biosecurity measures. Prophylactic/emergency vaccination may also be implemented, and is the main control measure used to control infection in endemic settings [3]. Virtually all FMD vaccines comprise chemically inactivated FMDV isolates [4]. Highly concentrated “banks” of this antigen are maintained by non-endemic countries, allowing rapid vaccine formulation in the event of an outbreak. The antigen chosen for formulation into a vaccine must be of a suitable potency and have broad antigenic cross reactivity with other isolates. Monitoring antigen cross-reactivity is essential to ensure current vaccines are effective against circulating strains. This is assessed using reference serum which has been raised against individual vaccine antigens [5], [6] and used in serological assays such as a 2-dimensional virus neutralisation test (2D-VNT) generating r_1_-values [7]. The r_1_-value is derived by dividing the heterologous neutralisation titre by the homologous neutralisation titre; for the VNT a value equal to or >0.3 suggests that the vaccine virus may impart adequate protection against the heterologous field strain under test [8]. Identification of an emergent virus with a poor (<0.3) r_1_-value against current vaccines may indicate that a new vaccine strain is needed.

However, vaccines with a poor r_1_-value have been shown to afford protection against heterologous challenge if the vaccine is of sufficiently high “potency” [9]. This potency is determined by undertaking tests prescribed by both the World Organization for Animal Health (OIE) section 5.3 [10], and the European Pharmacopoeia (Ph.Eur) Monograph 01/2017:0063 [11] and have historically always been carried out with the homologous virus for this purpose. There are two tests described: a protective dose (PD_50_) test and a protection against podal generalisation (PPG) test. One PD_50_ denotes the dose of a vaccine which would protect 50% of vaccinated animals. The quantity of PD_50_ doses present in a standard dose is its PD_50_ value. A PD_50_ of greater than or equal to 3 is classed as acceptable for routine vaccination, while a PD_50_ of greater than or equal to 6 is suitable for emergency vaccination. The PPG test gives the measure of potency in terms of the percentage of animals vaccinated with a single dose that are protected. A value of at least 75% is the pass level in the Ph.Eur and also considered epidemiologically appropriate [11], [12].

In 2015, samples submitted to the World Reference Laboratory for FMD (WRLFMD) as part of OIE/FAO global surveillance activities detected a new serotype A lineage (A/ASIA/G-VII) [13], [14]. This serotype A lineage emerged from the Indian sub-continent to cause FMD outbreaks in Saudi Arabia, Iran, Turkey, and Armenia. Four isolates of this A/ASIA/G-VII lineage were further antigenically characterised by 2D-VNT at the WRLFMD; the results of which became the provenance of this study. The objective of this study was to assess the heterologous performance of a multivalent vaccine, incorporating two serotype A components (A-SAU-95 and A-IRN-05), in affording protection of cattle from challenge with a currently circulating field virus of the A/ASIA/G-VII lineage.

## 2 Methods

### 2.1 Vaccine matching

Vaccine matching for 10 FMDV field isolates from the A/ASIA/G-VII lineage was elucidated by using a 2D-VNT with IB-RS-2 (renal swine) cells based on the original method [8] and outlined in the OIE Manual of Diagnostic Tests and Vaccines for Terrestrial Animals [15]. These field isolates were tested as part of the WRLFMD remit as an OIE/FAO reference laboratory. The details of some of these isolates have been published previously (13, 14); however, information regarding the provenance of some of the isolates is limited due to the lack of information received from the submitting countries.

These isolates were tested against a panel of eleven sera, each raised against a single type A vaccine antigen. The sera is derived from a high potency vaccine (at least 6PD_50_) and is a pool of five cattle which has been vaccinated with a monovalent vaccine. The sera was taken 21 days post vaccination. Briefly, the titres were calculated as the antibody dilution required to neutralise 50% of virus/cell mixtures at a virus dose of 100 Tissue Culture Infective Dose 50 (TCID_50_) and presented as the reciprocal. The 100 TCID_50_ was obtained by using five virus doses spanning from 10 to 1000 TCID_50_; each of these viruses doses were tested against a serial twofold dilution of sera. Using linear regression, the expected neutralisation at 100 TCID_50_ was derived.

The r_1_-values are calculated by taking the arithmetic mean of the field virus neutralisation titre and dividing it by the arithmetic mean of the vaccine virus neutralisation titre. A value equal or >0.3 suggests that a high potency vaccine will be protective against the field virus. Each r_1_-value is based on at least two sets of individual results.

### 2.2 Virus and vaccine

A virus isolate (A/IRN/22/2015) belonging to the FMDV A/ASIA/G-VII lineage was used to challenge the animals in this study. This challenge material was a 10% (w/v in M25 PBS) suspension of homogenised tongue lesion collected from Iran and titrated on bovine thyroid (BTY) cells [16] adjusted to a concentration of 2.5 x 10^5^ TCID_50_/ml. Titration on BTY cells has been shown to be 10x more sensitive than titration on bovine tongues (Bovine Infectious Dose 50 - BID_50_), and also more ethical [16], [17]. The inoculum titre was equivalent to 2.5 x 10^4^ BID_50_/ml.

A high potency (≥6PD_50_ per dose) batch of Aftovaxpur® vaccine containing each of the FMDV components; O_1_ Manisa, O-3039, O-PanAsia-2, A-IRN-05, A-SAU-95, SAT 2 and Asia-1-Shamir was prepared by Boehringer Ingelheim, Pirbright, United Kingdom, batch # A-415. The antigens were double inactivated (BEI), purified, with AI(OH)_3_ and saponin used as adjuvants. This vaccine was chosen because it represents the current vaccine being used in the region where A/ASIA/G-VII outbreaks were seen.

### 2.3 Vaccine challenge in cattle

All animal work was undertaken compliant with the Animals (Scientific Procedures) Act 1986, EU Directive 2010/63/EU, and licenced by The Home Office after local ethical review. The PPG test was carried out according to Ph.Eur monograph 01/2015:0063. The PPG test was chosen as the PD_50_ test suffers from a low level of reproducibility [18] in contrast to the PPG test which is considered more reliable in ascertaining vaccine efficacy [19].

Briefly, eighteen castrated male Holstein-Friesian cattle between 6 and 7 months of age (170–230 kg) were brought into the SAPO4/BSL3-Ag high-containment animal facility at TPI and group housed.

On day 0, 10 ml of blood were collected from each animal. Thereafter, 16 animals were each administered 2 ml of the vaccine via the sub-cutaneous route (1.5 in. needle). The remaining two cattle remained unvaccinated. Twenty-one days following vaccination (“day 21” or “0 days post challenge” (dpc)), 10ml of blood was collected then all 18 animals were sedated by administering a single intramuscular injection of xylazine (0.22 mg/kg). Upon ventral recumbency, 0.1 ml of the challenge virus was injected into the intradermal space of the tongue epithelium. This was repeated 3 more times in different areas of the tongue, a total of 10^4^ BID_50_. Sedation was reversed by administering the atipamezole (0.1 mg/kg) via the intravenous route. Every day thereafter 10 ml of blood, nasal swabs, and rectal temperatures were collected up to and including day 29 (8dpc). All animals were examined daily for clinical signs. Animals were culled at the following end-points: (i) the control animals developed lesions on at least three feet; (ii) the vaccinated animals developed lesions on at least one foot (unprotected), or (iii) the animals were culled on welfare grounds. On day 29 all animals remaining were culled. At the time of culling, irrespective of the day, detailed inspection of the feet was undertaken.

The PPG was calculated by dividing the number of vaccinated protected animals by the total number of vaccinated animals.

Serum samples collected at 0 and 21 days post vaccination were examined for anti-FMDV neutralising antibodies against the vaccine components and the challenge virus A/IRN/22/2015 using the protocol described above for vaccine matching.

### 2.4 Viraemia

RNA was extracted from serum using a MagMAX™ Express-96 Deep Well Magnetic Particle Processor (Thermal Fisher scientific, Waltham, USA) using the MagMAX™ -96 Viral RNA isolation kit (Thermal Fisher scientific, Waltham, USA) as per the manufacturer’s instructions. Viral RNA was quantified on a Mx3005P qPCR System (Agilent technologies, Santa Clara, USA) using an FMDV-specific qRT-PCR assay described previously [21] and a standard curve generated using 10-fold dilutions of an RNA. Viral RNA was quantified in copies/µl serum.

### 2.5 Nasal swabs

Nasal swabs were collected by swabbing the internal surface of both external nares in a circular motion with a single cotton swab. This swab was then placed in 2 ml Eagle’s Minimum Essential Medium (EMEM) supplemented with Amphotericin B, Penicillin, Neomycin and Polymixin B, vortexed to elute sample from the swab and stored at −80 °C. Plaque assays were carried out using a foetal goat tongue cell line (ZZ-R 127)[22]. Freshly prepared monolayers of cells in 6 well plates were infected with samples, overlaid with indubiose (Sigma) and incubated for 48 h. Virus was inactivated using citric acid, overlay removed, and cells stained using naphthol blue (Sigma).

### 2.6 Statistical analysis

The relationship between VNT and the probability of protection was analysed using generalised linear models with binomial errors and a logit link function. The response was status of the animal (i.e. lesions or no lesions) and the explanatory variable was log_10_ VNT. The analysis was carried out separately for each of two vaccine component viruses and for the challenge virus. Only vaccinated animals were included in these analyses.

To compare overall serum levels of FMDV the area under the curve (AUC; i.e. total amount of virus) was calculated by applying the trapezium rule to the time-course of copies/ml in serum for each animal. AUCs for the three groups of animals (i.e. unvaccinated, vaccinated-lesions, vaccinated-no lesions) were compared using a Kruskal-Wallis test, followed by pairwise Wilcoxon rank-sum tests to identify differences between groups.

Daily viral titres in nasal swabs were used to compute total shedding (i.e. AUC, calculated using the trapezium rule). Differences in total shedding between vaccinated animals of different status (i.e. vaccinated-lesions and vaccinated-no lesions) were assessed using Wilcoxon rank-sum tests. Correlation between total shedding and VNT titres (for each of the three serotype A viruses tested) was assessed used Spearman’s rank correlation coefficient.

## 3 Results

### 3.1 Vaccine matching

Vaccine matching results for field isolates from the A/ASIA/G-VII lineage are shown in Table 1. These data revealed poor antigenic match for all ten field viruses against serum raised to the eleven most commonly used FMDV serotype A vaccine antigens in this region, with all r_1_-values <0.3. The serum raised to three vaccine antigens (A-IRN-05, A-Iran-99, and A-Tur-14) showed no cross reactivity with any of the A/ASIA/G-VII isolates. These poor *in vitro* matching results, particularly those for A-IRN-05, suggests that current vaccines widely used in Asia may not be effective against viruses from this A/ASIA/G-VII lineage. Of the remaining 8 vaccine viruses, the highest level of cross reactivity was observed with the A-SAU-95. Therefore the purpose of this study was to assess whether the current high potency vaccine containing A-SAU-95 components vaccine could protect cattle against heterologous challenge with an A/ASIA/G-VII field isolate.

**Table 1:** Levels of cross-reactivity between a selection of antiserum raised against different vaccine antigens and ten A/ASIA/G-VII field isolates. These are given as both heterologous neutralisation titres (neut.) as the Log_10_ of the reciprocal titre, and serological relationship values (r_1_). r_1_ values equal to or above 0.3 suggest the there is a good match between the vaccine virus and the field isolate. < LDT = Lowest Dilution Titre - titre was below that of the first dilution tested.

	Vaccine virus																					
Field virus	A-IRN-05		A22-Iraq		A-MAY-97		A-TUR-20-06		A-SAU-95		A-IRN-87		A-IRN-96		A-IRN-99		A-IND-40-2000		A-TUR-11		A-TUR-14	
	neut.	r_1_	neut	r_1_	neut	r_1_	neut	r_1_	neut	r_1_	neut	r_1_	neut	r_1_	neut	r_1_	neut	r_1_	neut	r_1_	neut	r_1_
A/SAU/1/2015	<LDT	0	42	0.11	29	0.14	3	0.03	78	0.2	<LDT	0	19	0.04	<LDT	0	15	0.26	<LDT	0.01	<LDT	0
A/SAU/2/2015	<LDT	0	43	0.11	47	0.23	6	0.06	66	0.17	13	0.04	29	0.06	<LDT	0	not tested		not tested		not tested	
A/SAU/19/2016	<LDT	0	15	0.08	not tested		<LDT	0	33	0.22	not tested		not tested		not tested		not tested		not tested		not tested	
A/IRN/8/2015	<LDT	0	60	0.13	49	0.23	<LDT	0	102	0.26	not tested		not tested		not tested		not tested		not tested		not tested	
A/IRN/12/2015	<LDT	0	18	0.04	31	0.15	14	0.15	63	0.11	not tested		not tested		not tested		15	0.24	9	0.04	<LDT	0
A/IRN/22/2015	<LDT	0	27	0.2	not tested		<LDT	0	49	0.25	not tested		not tested		not tested		not tested		not tested		not tested	
A/IRN/25/2015	<LDT	0	<LDT	0	not tested		<LDT	0	not tested		not tested		not tested		not tested		not tested		not tested		not tested	
A/IRN/8/2016	<LDT	0	39	0.13	not tested		<LDT	0	not tested		not tested		not tested		not tested		3	0.03	<LDT	0.1	<LDT	0
A/SAU/24/2016	<LDT	0	21	0.12	not tested		<LDT	0	not tested		not tested		not tested		not tested		not tested		not tested		not tested	
A/SAU/41/2016	<LDT	0	39	0.22	not tested		<LDT	0	not tested		not tested		not tested		not tested		not tested		not tested		not tested	

### 3.2 Clinical signs

**Fig. 1:**
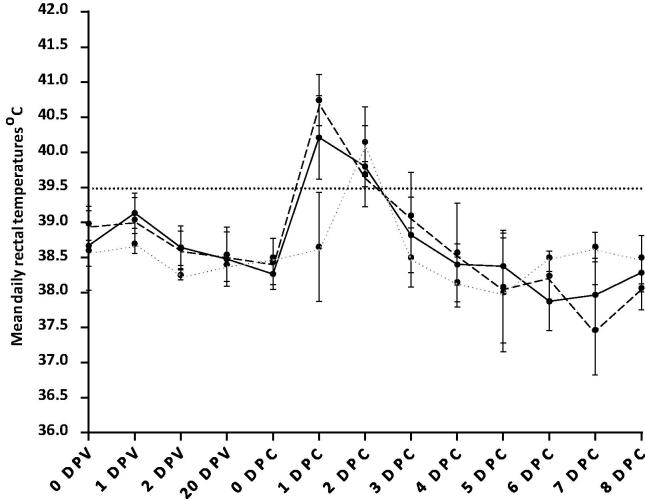
Mean daily rectal temperatures for all cattle before and after challenge with A/IRN/22/2015. Mean daily rectal temperatures for all cattle before (days post vaccination: DPV) and after challenge (days post challenge: DPC) with A/IRN/22/2015. Unvaccinated (⋅⋅⋅⋅⋅), vaccinated-lesions (− − −) and, vaccinated-no lesions (—) are all shown as separate line designs. Error bars represent the standard error. Horizontal dashed line is drawn at 39.5 °C; temperatures above which were classed as pyrexia.

After challenge all animals developed primary lesions at the site of inoculation. All animals displayed ptyalism, nasal discharge, and lip smacking. Seventeen out of the eighteen cattle had a rectal temperature over 39.5 °C (Fig. 1) and thus pyrexic [23] but pyrexia was not a reliable predictor of FMD generalisation, in agreement with previous studies [20], [24].

Both control animals developed vesicular lesions on at least three feet and seven out of sixteen vaccinates on at least one foot. The remaining nine vaccinated animals did not develop detectable lameness and were euthanized at the end of the experiment at 8dpc. Three animals were culled at 6dpc on welfare grounds. None of these animals had evidence of foot lesions. In total, 9 out of the 16 vaccinated animals were protected, equivalent to 56.3% PPG [95% confidence interval (CI): 33.2–76.6].

### 3.3 Serology

**Fig. 2:**
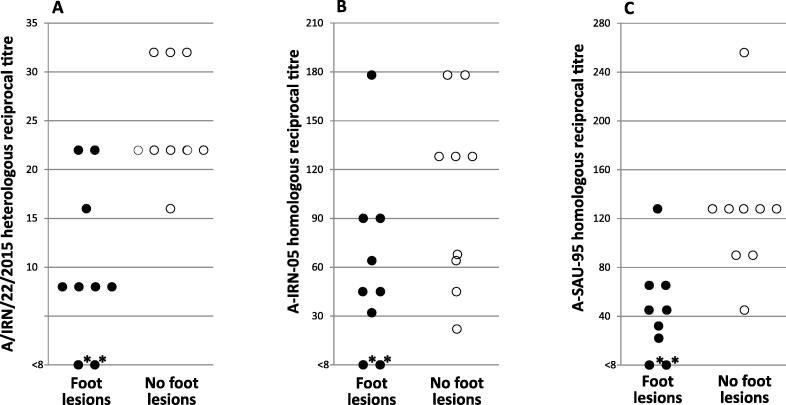
Serological responses on day 21 (day of challenge) of cattle given a full dose of the multivalent vaccine. Serological responses are displayed by plotting the reciprocal neutralising titre of each animal’s serum on the day of challenge when tested against the challenge virus (A) and each of the serotype A components of the vaccine (B and C). Animals are grouped into whether they developed foot lesions or no foot lesions. The two animals with foot lesions having a titre of <8 (denoted as*) are the two unvaccinated controls.

Table 2 shows the homologous serum titres from all animals at 21 days post vaccination. Prior to vaccination, no anti-FMDV antibodies were detected by VNT. On the day of challenge, the two unvaccinated animals remained seronegative, while all vaccinated animals developed titres measured against the individual vaccine components. Fig. 2 compares antibody titres of each animal against the 2 serotype A components of the vaccine and the challenge virus (A/IRN/22/2015).

**Table 2:** Reciprocal antibody titres of animal serum at 21 days post vaccination against each of the viruses listed. Reciprocal antibody titres in the serum of individual animals assayed on the day of challenge (21 days post vaccination) against each of the seven vaccine viruses (homologous) and the challenge virus A/IRN/22/2015. Titres given as <8 are assumed to be negative as 1/8 is the lowest titre which is possible to be detected with this assay. Animal IDs which are emboldened and underlined denote animals which proceeded to develop generalised disease and thus were unprotected.

Reciprocal antibody titres of animal serum at 21 days post vaccination against each of the viruses listed									
	Animal ID	A-IRN-05	A-SAU-95	Asia 1 Shamir	O 3039	O 5911	O Manisa	SAT 2 Eritrea	A/IRN/22/15
Vaccinated animals	3918	64	45	355	64	45	16	32	8
	**3920**	90	128	708	128	256	32	32	32
	**4910**	64	64	178	178	64	45	32	16
	4914	64	90	512	178	128	90	90	22
	4916	128	128	256	256	64	32	64	32
	**4917**	45	32	256	90	90	32	90	22
	**4921**	178	45	355	256	45	128	256	32
	4926	22	128	178	64	32	16	32	16
	**4930**	90	64	355	90	45	45	32	8
	**4932**	32	22	178	64	128	32	32	22
	4933	128	128	178	128	90	64	32	8
	4935	178	128	355	90	128	45	64	22
	4941	45	90	1024	128	178	45	64	22
	4942	178	128	355	355	90	128	45	22
	**4944**	45	45	128	45	32	45	22	22
	4945	128	256	355	90	45	64	64	8

Control animals	4919	<8	<8	<8	<8	<8	<8	<8	<8
	4939	<8	<8	<8	<8	<8	<8	<8	<8

As expected, the heterologous titres against the challenge virus (A/IRN/22/2015) were lower than the homologous titres against the two serotype A vaccine viruses. For the vaccine components, there was a significant increase in the probability of protection with increasing VNT for A-SAU-95 (p = 0.03), but not for A-IRN-05 (p = 0.42) as shown in Fig. 2. While a positive relationship was also observed for the challenge virus, this was found not to be statistically significant (p = 0.08).

### 3.4 Viraemia

**Fig. 3:**
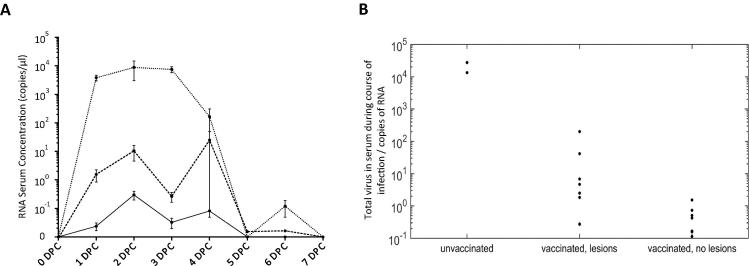
Graphs showing viral RNA levels over time in cattle challenged with A/IRN/22/2015. (A) Mean daily RNA concentrations in the serum for each time point for each of the 3 groups of animals (unvaccinated (⋅⋅⋅⋅⋅), vaccinated-lesions (− − − −) and, vaccinated-no lesions (—)). (B) Area under the curve (AUC) plotted for each animal, with animals grouped into the same 3 groups.

The daily concentration of FMDV RNA in the serum is shown in Fig. 3A. The animals with the highest peak levels of viraemia were the unvaccinated controls. Vaccinated animals had lower peak levels of viraemia after challenge. To examine the 3 groups of animals (naive, vaccinated protected, vaccinated unprotected) the area under the curve was calculated to represent total virus in serum over time (Fig. 3B). There was a significant difference in AUC amongst groups (Kruskal-Wallis test: P = 0.003). Pairwise comparison of the groups indicated a significant difference in AUC between the “unvaccinated” and “vaccinated without lesions” groups (Wilcoxon rank-sum test: P = 0.04) and between the “vaccinated with lesions” and “vaccinated without lesions” groups (Wilcoxon rank-sum test: P = 0.002), but not between the “unvaccinated” and “vaccinated with lesions” groups (Wilcoxon rank-sum test: P = 0.06).

### 3.5 Viral shedding from the nose

**Fig. 4:**
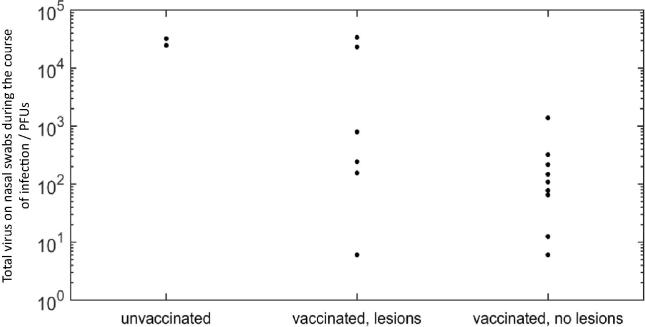
Graph showing quantities of live FMDV on nasal swabs taken daily from challenged animals. Total nasal shedding of FMDV over time. This is represented by calculating the area under the curve (AUC) for each animals daily FMDV shedding, and grouped into Vaccinated-no lesions, Vaccinated-lesions, and Unvaccinated.

Total virus shed from each animal was calculated by determining the area under each of the daily FMD virus shedding profiles. The highest levels of shedding were observed in the unvaccinated animals, with lower levels observed in the vaccinated animals (Fig. 4). The AUCs did not differ significantly (p = 0.09) between the “vaccinated with lesions” and “vaccinated without lesions” groups.

Total viral shedding was significantly (p = 0.02) negatively correlated (Spearman’s rho = −0.54) with virus neutralisation titre against A/IRAN/22/2015. A similar pattern was seen for the other two viruses tested but this was not statistically significant (p > 0.05).

## 4 Discussion

The vaccine used in this study is widely used in The Middle East and contains components which have been shown to match FMDV strains likely to be present in these regions. The poor *in vitro* matching data combined with anecdotal data describing outbreaks in vaccinated animals [13] suggested that the components in this vaccine may be ineffective against the A/ASIA/G-VII lineage. The experiment described here demonstrated that a high-potency poorly matched vaccine was able to afford protection against clinical FMD in individual animals, although the PPG value of 56.3% with the lower CI of 33.2% was below that described by both the OIE and European Pharmacopoeia. The OIE manual and European Pharmacopoeia suggests a 75% cut off for vaccine potency acceptance for those vaccines destined for use in regular vaccination regimens. In addition, the OIE manual outlining tests for the fitness of purpose of a routine vaccine suggests a 50% cut off for single dose vaccination, although other authors and the EPP method use a 75% cut off for fitness for purpose of a vaccine, which has been used with great success in South America [25].

The statistically significant correlation of increasing probability of protection with increasing antibody titres seen when using A-SAU-95 mirrors the r_1_ value data which supports the idea that only the A-SAU-95 component elicits antibodies which cross neutralise the A/IRN/22/2015 strain. Conversely, the poor correlation of increasing A-IRN-05 antibodies with protection (p = 0.42) supports the observed lack of cross reactivity of these antibodies with the challenge virus in the *in vitro* vaccine matching studies. Whilst a correlation between increasing titres of antibody to the challenge virus and probability of protection was observed, the lack of statistical significance is most likely due to the low numbers of animals used in the analysis, the overall lower titres of A/IRN22/2015 compared to the homologous A-SAU-95, and inherent variability in the VNT.

Each vaccine component was formulated >6PD_50_ by the vaccine manufacturer (equivalent to >90%PPG [26]), although this experiment did not use homologous challenge to specifically confirm this. The vaccine potency was assessed by using pre-existing serological correlates for each of the components with expected potency at the point of market authorisation. The varying reactivity of the antibodies in all animals at 21 days post vaccination suggest varying antigenic payloads in the vaccine, and is to be expected given formulation has been stated as containing a minimum of 6PD_50_ not precisely equal to 6PD_50_. Attempts to correlate VNT titre with protection and potency have been made, which allow vaccines to be assessed for potency without the need for challenge studies [27], [28]. The homologous titres for each of the 7 vaccine components observed were of a level that confirmed the high potency nature as reported by the vaccine manufacturer. The varying titres observed between the isolates are consistent with the observation that different serum titres of antibodies against different isolates are observed for a given *in vivo* potency.

Previous studies have shown that animals that are clinically protected against homologous challenge do not develop viraemia [29], [30]. In this study, quantitative real-time RT-PCR was used to show that 14/16 vaccinated animals developed viraemia. However, the results analysing the AUC showed that vaccination may reduce the total viraemic load over time when compared to non-vaccinated animals, irrespective of whether they were clinically protected or not. Use of the Kruskal-Wallis test to analyse difference between viraemia levels in the control animals and the vaccinated animals is valid given the test does not assume any distribution of data, but the results may not be robust given the small numbers of animals in the control group (two). There is no superior test to apply to this data, and so future studies using higher animal number to specifically address this observation may be necessary for more robust conclusions about the effect of vaccination on viraemia levels in unprotected animals. Moreover, the significant difference in total viraemic load between vaccinated protected and vaccinated unprotected animals, suggests reduced viraemia is either a cause of reduced generalisation or is a result of generalisation – a relationship yet to be elucidated. It has been shown, however, that levels of viraemia are an important marker associated with onward transmission and so the vaccine may be effective in reducing onward transmission, even in vaccinates which were not clinically affected [31]. As stated, whilst the PPG result along with its confidence intervals, is below that which is recommended, the viraemia data suggests this vaccine may reduce viral transmission even in clinically affected vaccinated animals [13]. In addition to viraemia, nasal shedding of FMDV has also been shown to correlate with onward transmission of FMDV from infected animals [31]. The reduction in nasal shedding imparted by the vaccine, even in vaccinated unprotected animals, again suggests that this vaccine may help reduce onward transmission of FMDV even if it does not afford clinical protection.

In summary, these data indicate that the multivalent vaccine tested in this study is not appropriate for emergency vaccination use against an incursion of A/ASIA/G-VII into previously FMD free countries. However, recent field data in a country where this vaccine is used, shows that higher homologous and heterologous titres may be achieved through frequent booster vaccinations [13], in line with previous studies that have investigated this phenomenon experimentally [32]. This study was performed directly in response to the initial reports of the emergence of the A/ASIA/G-VII lineage and utilised vaccine that was being used in field settings. A further monovalent heterologous challenge study may be warranted to confirm the results presented in this study with regard the sole efficacy of the A-SAU-95 component. These results may provide motivation for vaccine manufactures to develop a new vaccine strain with better cross reactivity with the A/ASIA/G-VII lineage.
